# Comparison of tissue acquisition techniques for Next-Generation Sequencing of non-small cell lung cancer (NSCLC)

**DOI:** 10.1136/bmjresp-2025-003793

**Published:** 2026-03-27

**Authors:** Amyn Bhamani, Sindhu Bhaarrati Naidu, Naimish Adroja, Lucy Rogers, Lavanya Anandan, Phil Bennett, Tanya Ahmad, Martin D Forster, Asia Ahmed, Ricky Thakrar, Sam M Janes, David A Moore, Neal Navani

**Affiliations:** 1 Lungs for Living Research Centre, UCL Respiratory, University College London, London, UK; 2 University College London Hospitals NHS Foundation Trust, London, UK; 3 Sarah Cannon Molecular Diagnostics, HCA Healthcare UK, London, UK; 4 CRUK Lung Cancer Centre of Excellence, UCL Cancer Institute, London, UK

**Keywords:** Lung Cancer

## Abstract

**Background:**

Next-Generation Sequencing (NGS) allows the use of more efficacious targeted treatments for lung cancer; however, sample inadequacy can cause delays in patient pathways. Here, we compare various methods of tissue acquisition used in clinical practice and identify factors associated with inadequate sampling.

**Materials and methods:**

Specimens submitted for NGS from a large single-centre UK academic institution following confirmation of lung cancer were reviewed. The primary objectives were to assess the proportion in which such analysis was successfully completed and identify factors, including the method of tissue acquisition, associated with sample inadequacy for, or failure of, the analysis. Secondary analyses included an assessment of genomic alterations identified by NGS-based analysis and specimen processing times.

**Results:**

DNA-based NGS analysis was successfully completed in 87.1% (n=511/587) of all specimens, with known oncogenic driver variants being identified in 60.6% (n=310/511). Success rates for specific specimen acquisition techniques included 90.0% (n=126/140) for endobronchial ultrasound-guided transbronchial needle aspiration, 79.3% (n=88/111) for percutaneous image-guided lung biopsies, 66.7% (n=14/21) for pleural fluid cell blocks and 60% (n=9/15) for percutaneous image-guided pleural biopsies.

Sequential RNA-based NGS analysis was successfully completed in 81.4% (n=92/113) cases, yielding a further 20 fusion gene events. Overall, actionable genomic alterations were identified in a total of 28.0% (n=143/511) of specimens across DNA-based and RNA-based analyses.

**Conclusion:**

Pleural fluid cell blocks and percutaneous image-guided pleural biopsies were least likely to be associated with successful NGS-based processing. Where possible, other sites for tissue acquisition should be considered in individuals with pleural disease to prevent delays in their pathway.

**WHAT IS ALREADY KNOWN ON THIS TOPIC**

- Next-Generation Sequencing (NGS) of non-small cell lung cancer (NSCLC) provides greater genetic information than single gene testing and European guidelines recommend that NGS is used routinely for advanced NSCLC.
- Some NGS technologies require greater DNA quality and quantity than PCR-based testing, and inadequate tissue sampling can delay patient pathways and increase risks associated with multiple biopsies.

**WHAT THIS STUDY ADDS**

- This study, the largest single-centre UK-based cohort to compare the adequacy of sampling techniques for NGS testing in NSCLC, demonstrates that pleural fluid cell blocks and non-surgical pleural biopsies are most frequently associated with inadequate sampling, and confirms that endobronchial ultrasound-guided transbronchial needle aspiration is a reliable method of tissue acquisition.
- NGS testing can be successfully integrated into routine clinical practice and can identify therapeutically actionable mutations in a high proportion of patients with NSCLC.

**HOW THIS STUDY MIGHT AFFECT RESEARCH, PRACTICE OR POLICY**

- Our data showing that non-surgical pleural samples are frequently inadequate for comprehensive biomarker testing should be considered by guideline committees when providing recommendations for pleural disease management.

## Introduction

 Lung cancer is the leading cause of cancer death in the UK^1^ and remains a condition that is predominantly diagnosed at late stage.^2^ Next-Generation Sequencing (NGS) encompasses a range of massively parallel DNA sequencing technologies that have revolutionised genomic testing by way of simultaneously capturing a much wider spectrum of genetic information than can be obtained from assessing single genes.^3^ In turn, this can enable the use of more efficacious targeted treatment strategies for individuals with advanced lung cancer and also reduce tissue requirements when compared with performing multiple less informative tests independently. In 2020, European Society for Medical Oncology guidelines recommended the routine use of NGS for the analysis of tumour samples in advanced non-squamous non-small cell lung cancer (NSCLC).^4^

Tissue NGS-based tests are typically associated with high sensitivity and specificity^5 6^ and a targeted DNA-based NGS panel has been shown to detect known oncogenic driver events in 65% of individuals with NSCLC.^7^ In addition, up to 26% of individuals with lung adenocarcinoma who previously tested negative for genomic alterations using non-NGS techniques have been found to have clinically relevant alterations on subsequent NGS-based testing.^8^ By enabling multiple target genes to be assessed simultaneously, NGS can also reduce the overall time to receive results that are fully informative with regards to therapeutic selection, as well as the need for retesting and/or repeat biopsies which may be associated with exclusionary sequential testing.^9^ Indeed, modelling undertaken in the USA suggests that NGS is associated with a lower overall cost and a significant reduction, of up to 4 weeks, in the time to initiation of appropriate targeted therapy, when compared with using sequential PCR-based testing strategies.^10^

The treatment landscape for NSCLC has rapidly evolved since the identification of gefitinib as targeted therapy for individuals harbouring EGFR mutations in 2004.^11^ In the UK, therapies targeting oncogenic variants in genes including anaplastic lymphoma kinase (ALK), BRAF, EGFR, KRAS, MET, NTRK, ROS1 and RET are now available in routine clinical practice.^12^ In addition, therapies for additional mutations such as ERBB2 (HER2)^13^ have been approved for use in the European Union and the USA, while new targets and associated targeted therapies are currently under evaluation in clinical trials. Consequently, NGS is likely to play an increasingly important role as we move further forward in the era of precision medicine.

Routine clinical practice incorporates a range of tissue acquisition techniques with overlapping roles in lung cancer management. For instance, endobronchial and peripheral lung biopsies are primarily undertaken for histological confirmation of malignancy, while pleural fluid aspiration and biopsies of pleura and extra-thoracic tissue (eg, peripheral lymph nodes or distant metastases) also help inform clinical staging. Pleural aspiration may also provide symptomatic relief to patients with large effusions. Similarly, endobronchial ultrasound-guided transbronchial needle aspiration (EBUS-TBNA) is also essential to accurately stage the mediastinum and support decision making regarding suitability for surgical resection and neo-adjuvant treatment. Finally, surgical resection remains the gold standard treatment for early-stage lung cancer, but also provides definitive diagnostic confirmation of malignancy when lesions are unsuitable for preoperative biopsy due to size or location.

These factors must be considered by lung cancer multidisciplinary teams (MDTs) when planning tissue acquisition for lung cancer. However, as some NGS technologies require a higher quality and concentration or even greater total DNA input than quantitative PCR, it is imperative that this is also considered when planning tissue acquisition to ensure that samples sufficient to facilitate successful NGS processing are obtained. However, data on the suitability of various tissue acquisition techniques used in clinical practice for NGS are limited.

A pathway for NGS and fusion gene testing implemented at our institution has previously been described.^14^ In this follow-up analysis, we assessed the adequacy of a range of biopsy techniques used in clinical practice for NGS and identified novel factors associated with inadequate sampling.

## Materials and methods

All cases of non-squamous NSCLC diagnosed at our institution are submitted for DNA-based NGS (DNA-NGS) analysis as routine standard of care. Testing was initially performed using Multi-Gene-Panel-1 (MGP-1) until mid-2020, when this was changed to an expanded Multi-Gene-Panel-4 (MGP-4).^15^ Both panels were ISO accredited to the international standard 15 189 and included EGFR, KRAS, BRAF, NRAS and ERBB2 as well as other less commonly mutated genes. Before 2021, all cases in which DNA-NGS was uninformative were also reassessed, and those suitable for further processing based on the amount of residual tumour tissue were submitted for fusion gene analysis by RNA-based analysis (RNA-NGS). The ISO-15189 scope of this fusion assay was restricted to inter-genic or intra-genic rearrangement involving ALK, BRAF, EGFR, MET, ROS1, RET, NTRK1 and NTRK3.

Percutaneous image-guided biopsies, including pleural biopsies, were primarily performed by three consultant thoracic radiologists cumulatively undertaking, on average, over 120 peripheral lung and 35 pleural or chest wall soft tissue biopsies per year. Biopsies were performed under CT or ultrasound (US) guidance using semi-automated spring activated cutting devices and 18G needles. Between three and eight passes were typically obtained per procedure. Percutaneous pleural biopsies included both targeted and non-targeted procedures. EBUS-TBNAs and bronchoscopies were performed by seven consultant respiratory physicians. Between four and six passes per lymph node were typically obtained during each EBUS procedure using 21G or 22G needles. Intranodal forceps and cryobiopsies were not used for EBUS procedures during the study period.

Specimens obtained via percutaneous image-guided biopsy, surgery and EBUS-TBNA were placed directly in formalin and processed for histological analysis. Pleural fluid specimens were sent fresh to the laboratory and processed onto slides using a 95% ethyl alcohol fixative to make the initial diagnosis. Following this, a formalin-fixed cell block was made from the fluid to facilitate further testing. All histology and cytology specimens were reviewed by consultant thoracic pathologists. Rapid onsite evaluation was not available.

We carried out a retrospective observational study of specimens submitted for NGS analysis, following confirmation of a lung cancer diagnosis, between 1 January 2018 and 31 December 2020. Specimens submitted for rapid PCR-based EGFR mutation analysis only (either on account of high clinical urgency or low DNA concentration) were excluded from the analysis. The primary objective was to assess the proportion of specimens in which DNA-based NGS analysis was successfully completed and identify factors, including the method of tissue acquisition and stage of disease, associated with inadequate sampling. The stage of disease was obtained from lung cancer MDT documentation (based either on pathological confirmation or by radiological consensus) at the time of biopsy. This included previously obtained surgical specimens submitted for NGS analysis in individuals subsequently presenting with recurrent disease. Missing stage was analysed as a separate category.

Secondary analyses included assessing the number of specimens in which known oncogenic driver mutations were identified and the processing time for each specimen. The latter was defined as the number of working days between specimen receipt in the molecular diagnostics laboratory and the date that the report was issued. The date of specimen receipt was termed ‘Day 0’.

Multivariable logistic regression analysis was used to explore factors associated with a successful analytical outcome. In addition to the method of tissue acquisition, the multivariable analysis was adjusted for patient and specimen age (defined as the number of days between biopsy and specimen receipt in the molecular diagnostic laboratory) as continuous variables, sex, disease stage and type of NGS panel. Complete case (listwise) analysis was used where the method of tissue acquisition was missing. χ^2^ analysis was used to compare differences between groups with categorical variables. A p value of <0.05 was considered significant. Statistical analyses were performed using R (V 4.4.1).

Full ethics committee approval was not required, as confirmed by the National Health Service (NHS) Health Research Authority decision tool.^16^ Informed patient consent was not obtained due to the retrospective, observational and non-interventional nature of the study.

### Patient and public involvement

Patients and members of the public were not involved in the design, conduct, analysis or dissemination of this study.

## Results

We reviewed 647 specimens from 560 individuals with NSCLC. 60 specimens underwent rapid PCR-based EGFR only testing on account of high clinical urgency and were excluded from subsequent analysis, meaning that 587 were included in the final analysis.

### Baseline demographic and clinical data

Median patient age was 69.0 years (IQR 58.0–75.0) with 52.5% (n=308/587) of specimens obtained from men. Most specimens were from individuals aged ≥60 years (71.9%, n=422/587) and those with late stage (3 or 4) disease at the time of biopsy (73.6%, n=432/587). Staging was not available in a minority (7.7%, n=45/587) of cases (table 1).

**Table 1:** Demographic and clinical background of specimens submitted for DNA-NGS analysis

	Frequency (n)	Percentage (%)
Sex		
Female	279	47.5
Male	308	52.5
Median patient age (years)*	69.0 (IQR 58.0–75.0)	
Median specimen age (days)†	11.0 (IQR 8.0–18.0)	
Age groups*		
<50	52	8.9
50–59	113	19.3
60–69	148	25.2
70–79	200	34.1
≥80	74	12.6
Stage*		
1	83	14.1
2	27	4.6
3	111	18.9
4	321	54.7
Missing	45	7.7
Type of lung cancer		
Adenocarcinoma	535	91.1
Non-small cell carcinoma, not otherwise specified (NSCLC—NOS)	15	2.6
Squamous cell carcinoma	13	2.2
Large cell neuroendocrine (NEC)	9	1.5
Other‡	15	2.6
Type of specimen		
Flexible bronchoscopy§	40	6.8
EBUS-TBNA	140	23.9
Percutaneous image-guided pleural biopsy¶	15	2.6
Percutaneous image-guided extra thoracic tissue and lymph node biopsy	73	12.4
Percutaneous image-guided peripheral lung and mediastinal mass biopsy**	111	18.9
Other††	29	4.9
Pleural fluid cell block	21	3.6
Thoracic surgery—pleural biopsy	56	9.5
Thoracic surgery—lung and lymph node specimens‡‡	96	16.4
Missing	6	1.0
NGS panel		
MGP-1	425	72.4
MGP-4	162	27.6

* Age and stage of disease at the time of biopsy.

† Days between biopsy and specimen receipt in the molecular diagnostic laboratory.

‡ Other includes adenoid cystic carcinoma, adenosquamous carcinoma, carcinoid, poorly differentiated carcinoma, carcinosarcoma, combined adenocarcinoma and small cell carcinoma, combined large and small cell carcinoma, large cell carcinoma, myoepithelial carcinoma and sarcomatoid carcinoma.

§ Includes three radial EBUS.

¶ Includes one parasternal mass and two chest wall masses arising from the pleura.

** Includes two mediastinal masses and one chest wall mass.

†† Other includes samples obtained via neurosurgery, adrenalectomy, pericardial aspiration, uterine surgery, colonoscopy and lumbar puncture.

‡‡ Includes six mediastinal lymph node specimens obtained via cervical mediastinoscopy.

EBUS-TBNA, endobronchial ultrasound-guided transbronchial needle aspiration; MGP, Multi-Gene-Panel; NEC, neuroendocrine; NGS, next generation sequencing; NOS, not otherwise specified; NSCLC, non-small cell lung cancer.

EBUS-TBNAs were the most common method of tissue acquisition (23.9%, n=140/587) followed by percutaneous image-guided (CT or US) lung and mediastinal mass biopsies (18.9%, n=111/587) and thoracic surgical specimens (16.4%, n=96/587).

### Association between tissue acquisition technique and the likelihood of successful DNA-based NGS analysis

**Figure 1:**
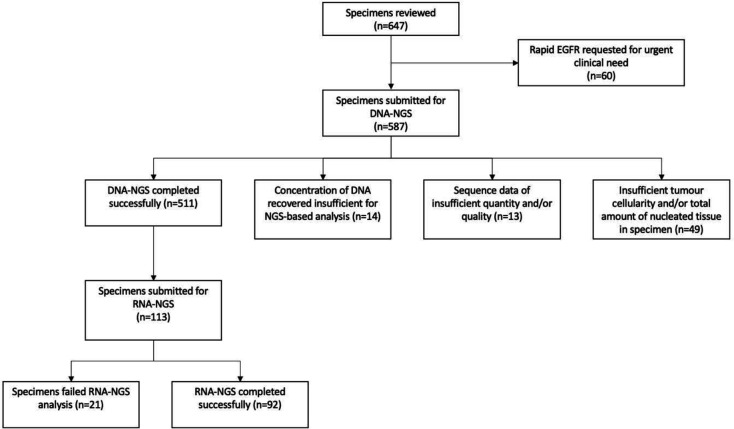
Summary of specimens reviewed and submitted for next generation sequencing. NGS, next generation sequencing.

DNA-based NGS analysis was successfully completed in 87.1% (n=511/587) of cases, with 8.3% (n=49/590) of specimens rejected outright due to insufficient tumour cellularity and/or the total amount of nucleated tissue in the sample. After fluorometric quantitation, the concentration of DNA recovered from a further 2.4% (n=14/587) of specimens that were initially accepted for processing was found to be insufficient for NGS-based analysis, and where possible, these were submitted for PCR-based EGFR testing. Finally, 2.2% (n=13/587) of specimens proved refractory to DNA-based NGS analysis, yielding sequence data of an insufficient quantity and/or quality. Consequently, DNA-based NGS analysis could not be completed in a total of 12.9% (n=76/587) of specimens overall (figure 1). The mean turnaround time in the molecular diagnostics lab for samples processed successfully was 5.9 working days from date of specimen receipt to date of report (SD 1.6).

Pleural fluid cell blocks and percutaneous image-guided pleural biopsies were associated with the lowest rate of analytical success (66.7%, n=14/21 and 60.0%, n=9/15, respectively, table 2). By contrast, DNA-based NGS analysis was successfully completed in 97.9% (n=94/96) of specimens obtained via thoracic surgery, 90.0% (n=126/140) obtained via EBUS-TBNA and 79.3% (n=88/111) obtained via percutaneous image-guided lung biopsy. Multivariable analysis (table 2) showed that these differences were statistically significant (table 2).

**Table 2:** Demographic and clinical factors associated with successful completion of DNA-based NGS assay

	Total specimens (n)	DNA-based NGS successful (%)	DNA-based NGS successful (aOR, 95% CI)	P value	DNA-based NGS successful (aOR, 95% CI)	P value
Sex						
Female	279	237 (84.9)	0.700 (0.431 to 1.137)	0.149	0.734 (0.436 to 1.237)	0.246
Male	308	274 (89.0)	1.00	–	1.00	–
Patient’s age*						
Per increasing year	–	–	0.982 (0.962 to 1.003)	0.090	0.986 (0.964 to 1.007)	0.193
Specimen age†						
Per increasing day	–	–	1.002 (0.998 to 1.005)	0.334	0.999 (0.996 to 1.003)	0.583
Stage*						
Missing	45	39 (86.7)	1.087 (0.436 to 2.713)	0.858	0.720 (0.256 to 2.024)	0.534
1	83	71 (85.5)	0.990 (0.498 to 1.967)	0.976	1.102 (0.416 to 2.918)	0.844
2	27	25 (92.6)	2.091 (0.479 to 9.128)	0.327	1.002 (0.179 to 5.606)	0.998
3	111	101 (91.0)	1.689 (0.822 to 3.474)	0.154	1.001 (0.429 to 2.337)	0.997
4	321	275 (85.7)	1.00	–	1.00	–
Type of specimen						
Flexible bronchoscopy	40	36 (90.0)	1.000 (0.310 to 3.226)	1.000	0.936 (0.287 to 3.054)	0.913
Percutaneous image-guided—pleural biopsy	15	9 (60.0)	**0.167 (0.052 to 0.538)**	**0.003**	**0.167 (0.049 to 0.569)**	**0.004**
Percutaneous image-guided—extra thoracic tissue and lymph node	73	63 (86.3)	0.700 (0.294 to 1.664)	0.420	0.719 (0.283 to 1.829)	0.489
Percutaneous image-guided—peripheral lung and mediastinal mass	111	88 (79.3)	**0.425 (0.207 to 0.872)**	**0.020**	0.432 (0.176 to 1.062)	0.068
Other	29	25 (86.2)	0.694 (0.211 to 2.285)	0.549	0.645 (0.181 to 2.295)	0.498
Pleural fluid cell block	21	14 (66.7)	**0.222 (0.077 to 0.643)**	**0.006**	**0.253 (0.077 to 0.830)**	**0.023**
Thoracic surgery—pleural biopsy	56	50 (89.3)	0.926 (0.337 to 2.545)	0.881	0.905 (0.303 to 2.706)	0.858
Thoracic surgery—lung and lymph node	96	94 (97.9)	**5.222 (1.159 to 23.533)**	**0.031**	**5.405 (1.043 to 28.001)**	**0.044**
Endobronchial ultrasound	140	126 (90.0)	1.00	–	1.00	–
Missing	6	6 (100)	–	–	–	
NGS panel						
MGP-4	162	144 (88.9)	1.264 (0.720 to 2.220)	0.414	1.273 (0.680 to 2.383)	0.451
MGP-1	425	367 (86.4)	1.00	–	1.00	–

Values in bold are statistically significant.

* Patient age and stage of disease at the time of biopsy.

† Specimen age was the number of days between biopsy and specimen receipt in the molecular diagnostics laboratory.

aOR, adjusted OR; MGP, Multi-Gene-Panel; NGS, next generation sequencing.

No significant associations were identified between analytical failure and sex, participant or specimen age, stage of disease at the time of tissue acquisition or type of NGS panel.

### Association between percentage tumour cellularity and the likelihood of successful DNA-based NGS analysis

Specimens with a histologically estimated tumour cellularity below the assay’s validated limit of detection (LOD) of 5% were considered unsuitable and rejected in all cases. DNA-based NGS analysis was completed successfully in all 15 (100%) specimens with a tumour cellularity >75%, and while lower, there was no significant difference (p=0.52) in likelihood of analytical success between specimens with tumour cellularities in the following ranges: 5%–20% (92.0% success), 21%–50% (93.1% success) and 51%–75% (95.2% success) (online supplemental table 1).

**Figure 2:**
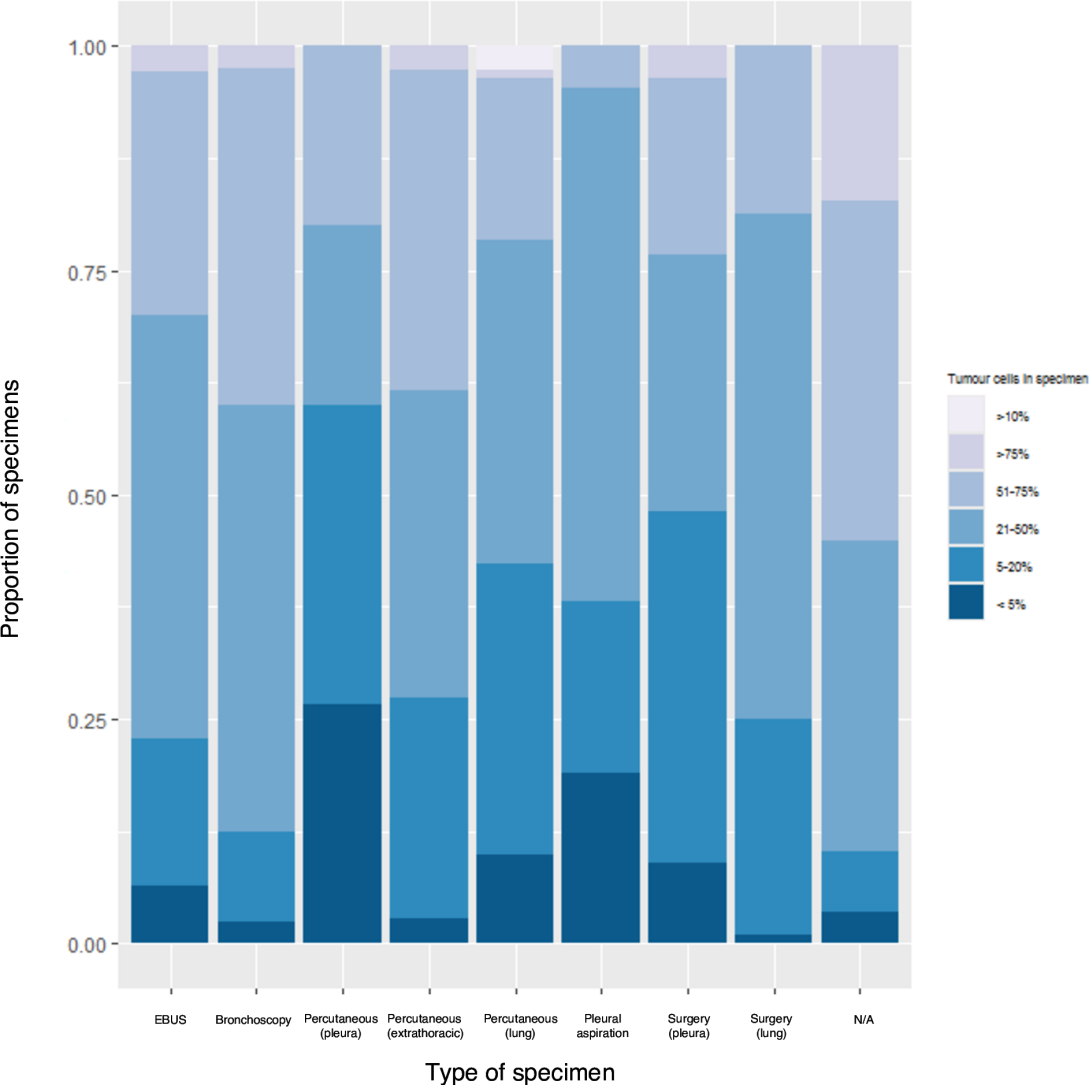
Percentage of tumour cells within specimen, stratified by tissue acquisition technique. EBUS, endobronchial ultrasound.

Percutaneous image-guided pleural biopsies (26.7%, n=4/15) and pleural fluid cell blocks (19.1%, n=4/21) were the collection techniques most frequently associated with the lowest tumour cellularity (<5%). In comparison, only 8.9% (n=5/56) of surgical pleural biopsies were associated with the lowest tumour cellularity (figure 2).

### Mutations identified by DNA-based NGS analysis

DNA-based NGS analysis identified known oncogenic driver variants in 60.6% (n=310/511) of specimens. KRAS mutations specifically were identified in 34.2% (n=175/511), including 10.6% (n=54/511) with the now actionable G12C variant and 0.4% (n=2/511) with dual mutations (KRAS A11T/G12V and KRAS G12V/G13) (table 3).

**Table 3:** Most common and clinically relevant mutations identified on DNA-NGS analysis

	Total, n (%)	Co-mutation with TP53, n (%)
KRAS (total specimens)	**175 (34.2)**	**74 (14.5)**
KRAS—G12C	54 (10.6)	23 (4.5)
KRAS—dual mutations	2 (<1.0)	2 (<1.0)
EGFR (total specimens)	**70 (13.7)**	**36 (7.0)**
EGFR—exon 19 deletions	31 (6.1)	17 (3.3)
EGFR—exon 19 single nucleotide variant	1 (<1.0)	0 (0.0)
EGFR—exon 20 insertions	2 (<1.0)	0 (0.0)
EGFR—exon 20 single nucleotide variant	7 (1.4)	3 (<1.0)
EGFR—exon 21 single nucleotide variant	32 (6.3)	17 (3.3)
EGFR—other mutations	5 (1.0)	3 (<1.0)
EGFR—dual mutations	9 (1.8)	4 (<1.0)
BRAF	**18 (3.5)**	**5 (1.0)**
ERBB2 (HER2)	**11 (2.2)**	**8 (1.6)**
MET (total specimens)	**9 (1.8)**	**4 (<1.0)**
MET (exon 14 skipping mutations)	3 (1.0)	2 (<1.0)
MET—dual mutations	1 (<1.0)	1 (<1.0)
STK11	17 (3.3)	10 (2.0)

Values in bold are statistically significant.

NGS, next generation sequencing.

EGFR mutations were identified in 13.7% (n=70/511) of specimens, with 13.3% (n=68/511) considered targetable with EGFR-tyrosine kinase inhibitors (TKIs) (online supplemental table 2). Dual EGFR mutations were reported in 1.8% (n=9/511). Exon 21 mutations were most common (6.3%, n=32/511), followed by exon 19 deletions (6.1%, n=31/511). Other, less commonly identified mutations included BRAF (3.5%, n=18/511), ERBB2 (HER2) (2.2%, n=11/511) and MET (1.8%, n=9/511). The MET aberrations included single nucleotide variants and three exon 14 skipping mutations associated with increased sensitivity to selected MET inhibitors. STK11 mutations were identified in 17 (3.3%) specimens—this included three (<1%) with KRAS/ STK11 co-mutations.

### Structural variants identified by RNA-based NGS analysis

Following uninformative DNA-based NGS analysis, 113 specimens were reflexed for RNA-based NGS analysis in an attempt to identify inter-genic and intra-genic fusions. 21 (18.6%) of these were unsuccessful, 1 being rejected as the remaining nucleated tissue had cut out during prior DNA extraction, 17 being rejected as unsuitable for processing due to insufficient RNA quality/quantity, and 3 which yielded inadequate sequencing data for any meaningful analysis. Of the remaining 92 specimens, 20 fusion events were identified in 19 samples, including six involving MET Exon 14 skipping and five involving ALK-EML4 fusion. Four fusions involving ROS1 were identified (online supplemental table 3).

Overall, known oncogenic driver variants were identified in 63.8% (n=326/511) of specimens following both DNA and reflex RNA-based NGS analyses, with 28.0% (n=143/511) of these being considered currently therapeutically actionable—this includes ALK fusion, BRAF V600E, KRAS G12C, MET-Exon 14 skipping, RET fusion, ROS-1 fusion and TKI-sensitive EGFR mutations.^17^

## Discussion

In this analysis of 587 specimens submitted for NGS analysis following confirmation of NSCLC, DNA-based NGS was successfully completed in 87% of cases. DNA-based NGS could not be completed in 13% of cases due to low tumour cellularity, insufficient total nucleated tissue, inadequate DNA yield (concentration/integrity) or actual NGS assay failure, likely due to additional DNA quality issues. Pleural fluid cell blocks (67%) and percutaneous image-guided pleural biopsies (60%) were associated with the lowest success rate, with 19% and 27% of specimens obtained via these techniques, respectively, rejected outright due to insufficient tumour cellularity. As expected, surgical lung and lymph node specimens were associated with the highest success rate, with only 2% unable to be analysed successfully.

Guidelines in the UK recommend pleural fluid analysis as the first line investigation for individuals presenting with suspected secondary pleural malignancy.^18^ However, a national study of molecular testing found that individuals with late-stage lung cancer who underwent pleural aspiration or biopsy were more likely to require a second biopsy compared with those who had an EBUS-TBNA.^19^ In keeping with these results, DNA-based NGS analysis was successful in only two-thirds of pleural fluid specimens in our series. In addition, while details on the number of patients with negative pleural fluid cytology prior to confirmation of lung cancer via alternative methods of specimen acquisition were not available for analysis, the sensitivity of pleural fluid cytology for diagnosing a malignant pleural effusion (MPE) in lung cancer is reported to be 62%.^20^ Extrapolating our results to these data suggests that only about 40% of pleural fluid specimens obtained from individuals with lung cancer and MPE would yield both a diagnosis and a cell block adequate for NGS-based analysis. Data from the USA also show that individuals requiring multiple biopsies may have up to 90 days added to their pre-treatment timeline on account of a significant time lag between each procedure, while multiple biopsies are associated with a higher incidence of complications such as pneumothorax and bleeding.^21^

Despite these considerations, it is important to acknowledge that pleural fluid analysis plays an important role in informing clinical lung cancer staging, particularly for patients with pleural effusions but an otherwise low probability of malignant pleural disease based on imaging features and clinical history. For these patients, negative pleural fluid cytology may enable them to have treatment with curative intent. However, timely initiation of treatment is also associated with a reduced risk of death among patients with lung cancer,^22^ and delays in NGS processing due to inadequate sampling may have a detrimental prognostic impact. Our data suggest that in otherwise well patients with a high probability of malignant pleural disease, pleural aspiration should be carried out for therapeutic purposes where possible, but referral for further specimen acquisition should not be delayed while awaiting the outcome of pleural fluid analysis. In such cases, an early referral for surgical pleural biopsy or local anaesthetic thoracoscopy, when more easily accessible sites for tissue acquisition are not present, would also allow definitive pleural fluid management (eg, in the form of pleurodesis or the insertion of a tunnelled indwelling pleural catheter) in the same sitting.

Over the past few years, EBUS-TBNA has become an increasingly accessible and important method of tissue acquisition for both diagnostic and staging purposes in the context of suspected lung cancer. While there have previously been concerns about the suitability of tissue acquired via this technique for subsequent NGS-based analysis, a recent meta-analysis of 1175 participants showed EBUS-TBNA was associated with a yield of 86.5% for NGS.^23^ In our cohort, 90% of specimens obtained via EBUS-TBNA were adequate for DNA-based NGS analysis, confirming the importance of this technique in the context of lung cancer diagnostics.

Underscoring the importance of NGS in ensuring the optimum treatment for individuals with lung cancer, 28% of specimens had actionable genomic alterations following both DNA and reflex RNA-based NGS analysis. Over 50% of specimens with EGFR mutations also harboured TP53 co-mutations, a combination associated with poor outcomes in individuals treated with EGFR-directed TKIs.^24^ Similarly, seven cases identified co-existing STK11 and KRAS mutations, with STK11 alteration previously identified as the most prevalent genomic driver of primary resistance to PD-1 axis inhibitors in KRAS-mutant lung adenocarcinoma^25^ and shown to be associated with a poor prognosis.^26^

RNA-based NGS analysis was associated with a lower rate of success (82%) compared with DNA-based (87%), despite specimens with insufficient tumour cellularity having already been excluded. This was partially due to the sequential extraction strategy employed during the period studied—specifically, the recall ‘batch’ testing of RNA only if required following initial DNA testing^14^—which meant that some samples may have been several months old at the time of RNA analysis, increasing the chance of tissue being exhausted. RNA is also known to be more unstable and prone to degradation by both natural and artificial processes such as those used during tissue processing into FFPE material.^27 28^ With regard to the former, techniques for the combined extraction of both DNA and RNA together are available; however, previous work by the laboratory (data not shown) had revealed a slightly higher failure rate due to lower average RNA quality from such combined extractions. Considering this and other factors such as cost, a sequential strategy was retained.

Limitations of this study are recognised. These data are from a specialist thoracic oncology centre with expertise in tissue acquisition, processing and on-site molecular testing. Additionally, both the minimum acceptable tumour cellularity, based on the assay’s validated LOD, and the quality/total amount of input DNA required by different laboratories can vary significantly. The latter depends on the library generation methodology, sequencing platform and the size of the gene panel used. Consequently, these findings may not be directly applicable to other centres. On the other hand, this is an NHS-based clinical cohort with genuine representation across a large selection of individuals with NSCLC, unbiased by factors such as medical insurance coverage/reimbursement, which may affect the make-up of cohorts from countries with different healthcare systems. In addition, despite the retrospective nature of the study, it is the largest to date to examine the efficacy of sampling techniques for NGS, there were clear criteria for patient inclusion, consecutive patients were included and NGS testing was standardised. The number of percutaneous image-guided pleural biopsies and pleural fluid cell blocks included in this analysis was also relatively small, and both targeted and non-targeted pleural biopsies were included in the analysis. However, this reflects the low prevalence (approximately 15%) of MPEs at presentation among patients with NSCLC reported in international thoracic oncology cohorts.^29^

In conclusion, 87% of lung cancer specimens obtained via a range of tissue acquisition techniques and submitted for DNA-based NGS analysis were successfully processed. Pleural fluid cell blocks and percutaneous image-guided pleural biopsies were techniques least likely to result in success, and other sites for tissue acquisition could be considered in these individuals. NGS is increasingly vital to provide important therapeutic and prognostic information for individuals with lung cancer and can be delivered in routine clinical practice.

## Supplementary material

10.1136/bmjresp-2025-003793 online supplemental file 1

## Data Availability

Data are available upon reasonable request.
