# Supplementary material for: Comparison of tissue acquisition techniques for Next-Generation Sequencing of non-small cell lung cancer (NSCLC)
Source: BMJ Open Respir Res. 2026 Mar 27;13(1):e003793. doi: 10.1136/bmjresp-2025-003793 (PMC13034294; doi:10.1136/bmjresp-2025-003793)
Supplement: online supplemental file 1 [file bmjresp-13-1-s001.docx]

**Supplementary material**

**Supplementary table 1:** Proportion of specimens where DNA-based NGS analysis completed successfully, stratified by tumour cellularity

| Estimated  Tumour  Cellularity | Total Specimens | Successful analyses (%) | Reason analysis unsuccessful | N |
| --- | --- | --- | --- | --- |
| <5% | 38 | 0 (0%) | Rejected - Insufficient tumour cellularity | 38 |
| 5-20% | 137 | 126 (92.0%) | Rejected - Insufficient tumour cellularity or total nucleated tissue | 6 |
|  |  |  | Processed – Failed yield viable NGS data | 4 |
|  |  |  | DNA concentration too low for NGS, EGFR only assessed by qPCR | 1 |
| 21-50% | 247 | 230 (93.1%) | Rejected - Insufficient tumour cellularity or total nucleated tissue | 5 |
|  |  |  | Processed – Failed yield viable NGS data | 6 |
|  |  |  | DNA concentration too low for NGS, EGFR only assessed by qPCR | 6 |
| 51-75% | 147 | 140 (95.2%) | Processed – Failed yield viable NGS data | 2 |
|  |  |  | DNA concentration too low for NGS, EGFR only assessed by qPCR | 5 |
| >75% | 15 | 15 (100%) | N/A | 0 |
| >10%* | 3 | 0 (0.0%) | Processed – Failed yield viable NGS data | 1 |
|  |  |  | DNA concentration too low for NGS, EGFR only assessed by qPCR | 2 |

*Cellularity logged as >10% because rapid EGFR was performed on these samples as a salvage pathway after NGS failure. 10% is the minimum cellularity for which the rapid EGFR assay is validated

**Supplementary table 2:** List of EGFR mutations identified during DNA-NGS analysis

| **EGFR mutation** | **Type of mutation** | **Responder to TKI** | **Frequency (n)** |
| --- | --- | --- | --- |
| EGFR.*Glu709Ala  EGFR.*Gly719Ala  EGFR.*Gly719Cys | Exon 18 SNV | Yes | 1  1  2 |
| EGFR.*Glu709_Thr710delinsAsp | Exon 18 deletion | Yes | 1 |
| EGFR.*Leu747_Pro753delinsSer  EGFR.*Glu746_Ala750del  EGFR.*Leu747_Thr751del  EGFR.*Leu747_Ala750del  EGFR.*Ser752_IIe759del  EGFR.*Glu746_Thr751delinsAla  EGFR.*Thr751_lle759delinsAsn  EGFR.*Glu746_Ser752delinsVal | Exon 19 deletion | Yes | 4  16  3  2  1  3  1  1 |
| EGFR.*Ile744Met | Exon 19 SNV | Yes | 1 |
| EGFR.*Ser768_Asp770dup  EGFR.*Asp770_771insSerValAsp | Exon 20 insertion | Yes | 1  1 |
| EGFR.*Thr790Met  EGFR.*Ser768Ile | Exon 20 SNV | Yes | 4  2 |
| EGFR.*Val774Met  EGFR.*Arg776His | Exon 20 SNV | No | 1  1 |
| EGFR.*Leu858Arg  EGFR.*Leu861Gln | Exon 21 SNV | Yes | 30  1 |
| EGFR.*Ala871Thr | Exon 21 SNV | No | 1 |

**Supplementary table 3:** Summary of mutations identified across RNA-NGS analysis

| Fusion gene detected | Total number |
| --- | --- |
| MET - Exon 14 skipping | 6 (including one with RET rearrangement) |
| ALK - EML4 | 5 |
| EZR - ROS1 | 2 |
| CD74 - ROS1 | 1 |
| SDC4 - ROS1 | 1 |
| EGFR - ETV1 | 1 |
| RET rearrangement | 2 (including one with MET – Exon 14 skipping) |
| ALK rearrangement (NOS) | 1 |
| BAG4 - FGR1 | 1 |
